# A bibliometric analysis of the theme trends and knowledge structures of pulmonary embolism from 2017 to 2021

**DOI:** 10.3389/fmed.2023.1052928

**Published:** 2023-02-23

**Authors:** Jingjing Fan, Xiaoman Xu, Li Zhao

**Affiliations:** Department of Pulmonary and Critical Care Medicine, Shengjing Hospital of China Medical University, Shenyang, China

**Keywords:** pulmonary embolism, bibliometric analysis, hot research topics, theme trends, knowledge structures

## Abstract

**Background:**

Pulmonary embolism (PE) is a popular area of research in the field of respiratory medicine. We performed a bibliometric analysis based on PubMed database to characterize the distribution pattern of literature and knowledge structures related to PE.

**Methods:**

Literature pertaining to PE from January 1, 2017, to December 31, 2021, was retrieved from the PubMed database. Bibliographic information was generated using the Bibliographic Item Co-Occurrence Matrix Builder (BICOMB). The visualization matrix was established using gCLUTO software. Strategic diagram analysis was performed using GraphPad Prism 9 software. Social network analysis (SNA) was generated using Ucinet6.0 and NetDraw 2.084 software.

**Results:**

Out of all the retrieved MeSH terms and subheadings, 52 MeSH terms/MeSH subheadings with a high frequency were found, and hot subjects were sorted into 6 clusters. The strategy diagram showed that the epidemiology, etiology, and drug therapy of PE were well advanced. In contrast, studies on diagnostic imaging, pathology, and complications of PE were still immature and offered potential research space. Social network analysis showed that marginal topics such as surgical treatment of pulmonary hypertension, prevention and control of postoperative complications, and metabolism and analysis of fibrin/fibrinogen degradation products were emerging research hotspots.

**Conclusion:**

Objective analysis of the research developments in the field of PE can provide intuitive knowledge structure for researchers and clinicians. Analysis of the research hotspots related to PE is helpful for researchers and clinicians by highlighting future research directions.

## Introduction

Pulmonary embolism (PE) refers to an obstruction in the pulmonary artery due to a clot, tumor, air, or fat, and is a form of venous thromboembolism (VTE) (1, 2). The clinical spectrum of PE depends on the hemodynamic consequences and ranges from asymptomatic PE to life-threatening medical emergency (3). Globally, PE is the third leading cause of cardiovascular death after stroke and coronary artery disease (4).

In recent years, an extensive body of research related to PE has been published, mainly focusing on the epidemiology, etiology, diagnosis, treatment, and complications of PE. However, bibliometric studies on the structures and trends of PE have not been reported.

Bibliometrics refers to the quantitative analysis and interpretation of topical issues in the literature. Co-term analysis is one of the more commonly used methods in bibliometrics to estimate the heterogeneity and correlation of two groups of words in a knowledge network (5). In addition, this work applied biclustering analysis, strategy diagram, and social network analysis to analyze the relationships between themes as well as trends. From the perspective of bibliometrics, this work aimed to characterize the knowledge structure and research trend related to PE and provide broader ideas for researchers and clinicians for the future research directions related to this subject.

## Materials and methods

### Data collection

The US National Center for Biotechnology Information’s PubMed database was used to retrieve and download data. The National Library of Medicine’s Medical Subject Heading (MeSH) terms, a medical vocabulary resource, are used to index and categorize articles in the PubMed database. During retrieval of articles, the scope of retrieval was limited to journal articles (publication type), English (language of publication), and with no limitation of species. The retrieval term used was “PE” [MeSH]. The reference period for retrieval was from 1 January 2017 to 31 December 2021. A total of 5,583 items were retrieved. The following details were collected for each article: title, author, institution, country, publication year, and MeSH terms. These records were kept in TXT format.

### Data extraction and bibliographic matrix setup

BICOMB was used to extract and count bibliographic information accurately (6). The bibliographic information included journals, countries, years of publication, authors, and main MeSH terms/MeSH subheadings. The software was used to rank journals, years of publication, authors, and frequency of main MeSH terms/MeSH subheadings, and to record the frequency of main MeSH terms/MeSH subheadings.

In addition, the h-index was used to determine the amount of high-frequency main MeSH terms/MeSH subheadings. The *h*-index was proposed in 2005 by J. E. Hirsch, a physicist at the University of California at San Diego, United States. According to the definition of h-index, when intercepting high-frequency terms, the terms were first arranged in descending order of word frequency, and the terms with word frequency greater than or equal to their ordinal number (*h*) were searched for from the list of terms. The word frequencies of its subsequent term were less than h. The threshold value for intercepting high-frequency main MeSH terms/MeSH subheadings was set at *h* (7). Based on the ranking, the h-index of this study was 52, so there were 52 high-frequency main MeSH terms/MeSH subheadings, and these high-frequency main MeSH terms/MeSH subheadings, represented the research hotspots of PE. The term-source article matrix and co-occurrence matrix were built using BICOMB.

### Biclustering analysis of high-frequency main MeSH terms/MeSH subheadings

High-frequency main MeSH terms/MeSH subheadings were categorized using the term-source article matrix, and the high-frequency MeSH terms and PubMed unique identifiers from the retrieved literature on PE were used in the biclustering analysis. Using the repeated bisection method in the gCLUTO software, a visual matrix was produced after clustering the mountain visualization (8). The peaks in the 3-D terrain were labeled with numbers representing clusters analyzed by biclustering. Namely, the ordinal numbers of the clusters were automatically generated by the mountain visualization. The data for the relevant clusters were represented by the color, height, volume, and location of each peak in the figure. The height of each peak in a cluster determines its internal resemblance. The distance between two peaks indicates their relative resemblance. A peak’s volume is proportional to the number of main MeSH terms/MeSH subheadings. The color of the peak represents the internal standard deviation of the components of a cluster. Blue indicates high deviation and red indicates low deviation. On the left and top of the matrix, respectively, the row labels for the matrix representation denote the primary MeSH terms and MeSH subheadings, and the column labels denote the PubMed unique identifiers of the source articles. In addition, the software can generate clustering analysis solution results, which contain representative literature for each cluster. Combined with the main MeSH terms/MeSH subheadings of the row labels, the components of each cluster can be identified. The structure of the associated research focus was investigated using the findings of the biclustering analysis.

### Strategic diagram analysis

The strategic diagram was used to describe the external and internal cohesion of a research area. A strategic diagram was a two-dimensional diagram that was constructed by calculating the density and centrality of thematic clusters and plotting them along the *X*-axis and *Y*-axis (9). In the strategic diagram, the *X*-axis represents the centrality or external cohesion index, which represents the strength of interactions between themes, that is, their central position in the overall network. *Y*-axis represents the density or internal cohesion index, which represents the ability of themes to maintain and develop themselves (10). The detailed method for calculating the two parameters was described by Callon (11). There were two axes generating four quadrants to which the main MeSH terms/MeSH subheading clusters were assigned based on the results of the biclustering analysis. Strategic diagram was generated using Graph Pad 9 software (GraphPad Software Inc., San Diego, CA, United States).

### Social network analysis

Social network analysis was performed to analyze the knowledge structure of PE based on the co-occurrence matrix by constructing a network with Ucinet 6.0 (Analytic Technologies Co., Lexington, KY, United States) and NetDraw 2.084 software. The main MeSH terms/MeSH subheading of the network was represented by nodes and the co-occurrence frequency was represented by links. The degree, betweenness, and closeness centrality measures of each node were utilized to assess the positions of the main MeSH terms/MeSH subheadings in the network to comprehend the network structure of PE.

## Results

### Distribution features of relevant literature

**Figure 1:**
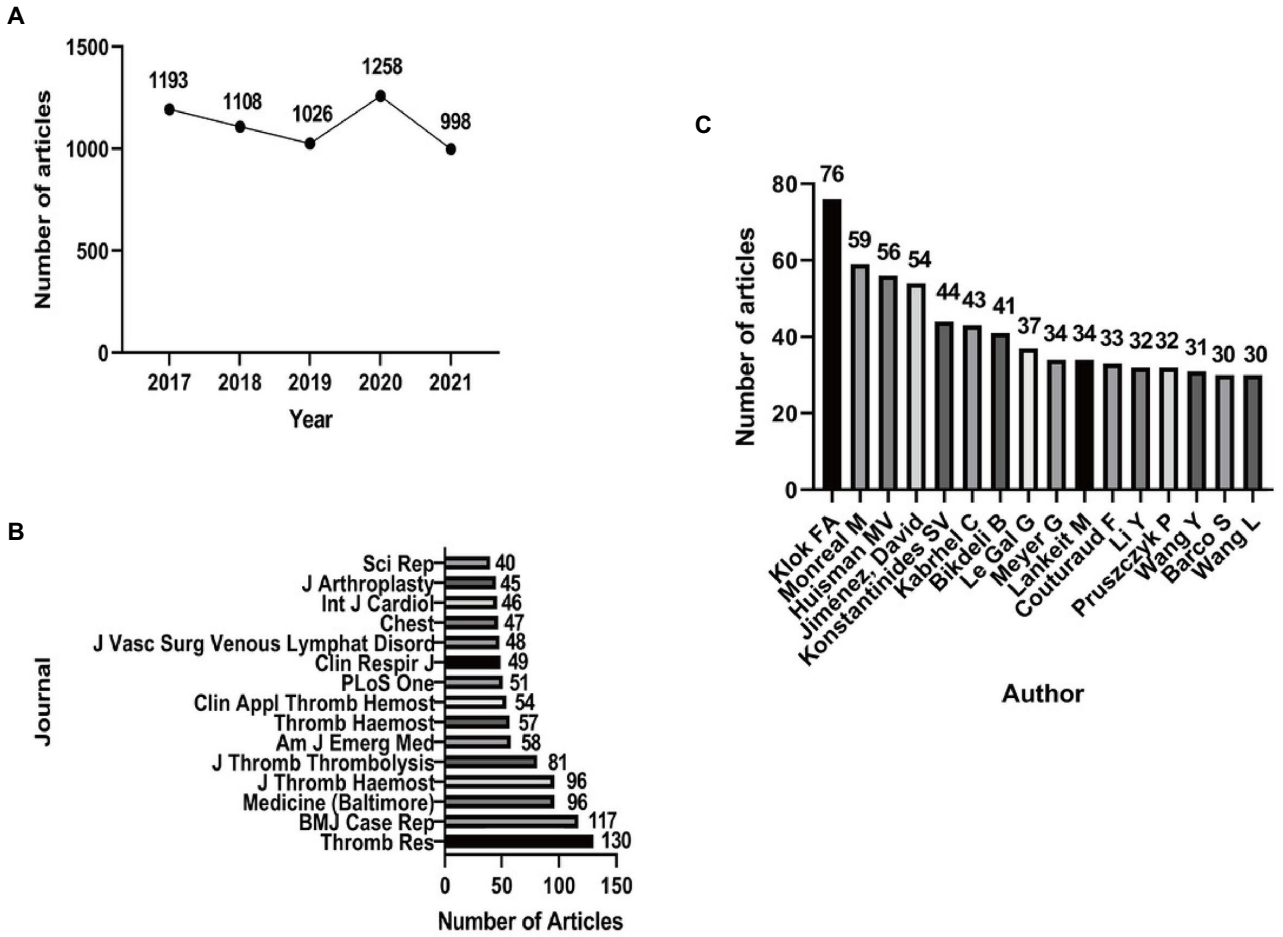
Features of PE-related publications. **(A)** The number of publications on PE from 2017 to 2021. **(B)** The number of publications in the top 15 journals. **(C)** The number of publications from the top 15 first authors.

From 2017 to 2021, there were a total of 5,583 publications that qualified the search criteria. The number of articles related to PE was 1,193 in 2017, decreasing slightly from 2018 to 2019, followed by a significant increase in 2020 (1,258) and decrease again in 2021 (998) (Figure 1A). In terms of journal ranking, Thromb Res published the highest number of relevant articles (130; 2.328%), followed by BMJ Case Rep (117; 2.096%), with Medicine (Baltimore) and J Thromb Haemost (96, 1.720%) tied for the third place (Figure 1B). The top 15 first authors in this field among the included articles are displayed in Figure 1C. Among the first authors, Klok FA had authored the most articles.

### Research hotspots concluded by MeSH term clusters

**Figure 2:**
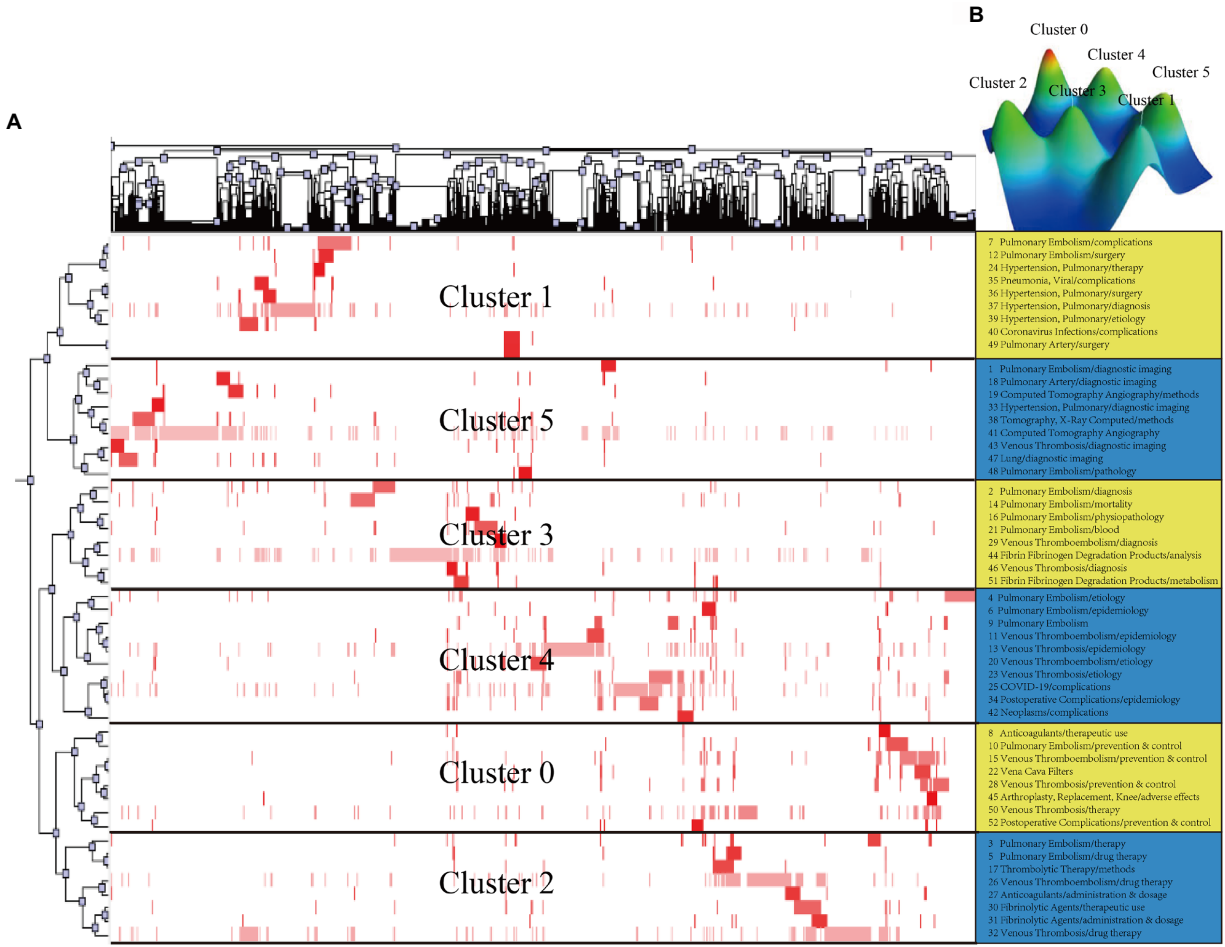
Biclustering analysis of 52 high-frequency main MeSH terms/MeSH subheadings and articles on PE from 2017 to 2021. **(A)** Matrix visualization of biclustering of 52 high-frequency main MeSH terms/MeSH subheadings and PubMed Unique Identifiers of the articles. **(B)** Mountain visualization of biclustering of 52 high-frequency main MeSH terms/MeSH subheadings and articles.

Fifty-two high-frequency main MeSH terms/MeSH subheadings with a combined frequency of 40.7497% were found in the literature on PE (Supplementary Additional Table 1). These could be regarded as the research hotspots in the previous 5 years. The above 52 high-frequency terms were divided into 6 clusters by biclustering analysis. The main MeSH terms/MeSH subheadings are visualized as mountains and matrices in Figure 2. The cluster trees on the left and the top indicate the relationship between main MeSH terms/MeSH subheadings and the relationship between main MeSH terms/MeSH subheadings and articles, respectively. The right side shows the 52 high-frequency main MeSH terms/MeSH subheadings, and the numbers in front of them indicate the ranking of the main MeSH terms/MeSH subheadings. Table 1 lists the findings of the cluster analysis performed on the high-frequency main MeSH terms/MeSH subheadings of studies relevant to PE.

**Table 1:** Cluster analysis of high-frequency main MeSH terms/subheadings of PE.

Cluster	Number of MeSH terms/MeSH subheadings*	Cluster analysis
0	8,10,15,22,28,45,50,52	1. Prevention and control of PE
		2. Prevention and control of postoperative complications
1	7,12,24,35,36,37,39,40,49	1. Complications of PE
		2. Surgery of pulmonary hypertension
2	3,5,17,26,27,30,31,32	1. Drug therapy of PE
		2. Drug therapy of VTE
3	2,14,16,21,29,44,46,51	1. Diagnosis of PE
		2. Diagnosis of VTE
		3. Analysis and metabolism of fibrin/fibrinogen degradation products
4	4,6,9,11,13,20,23,25,34,42	1. Epidemiology of PE and VTE
		2. Etiology of PE and VTE
5	1,18,19,33,38,41,43,47,48	1. Diagnostic imaging of PE
		2. Pathology of PE

*Represents rank of high-frequency main MeSH terms/subheadings shown in Supplementary Additional Table 1.

### Theme trends of PE

**Figure 3:**
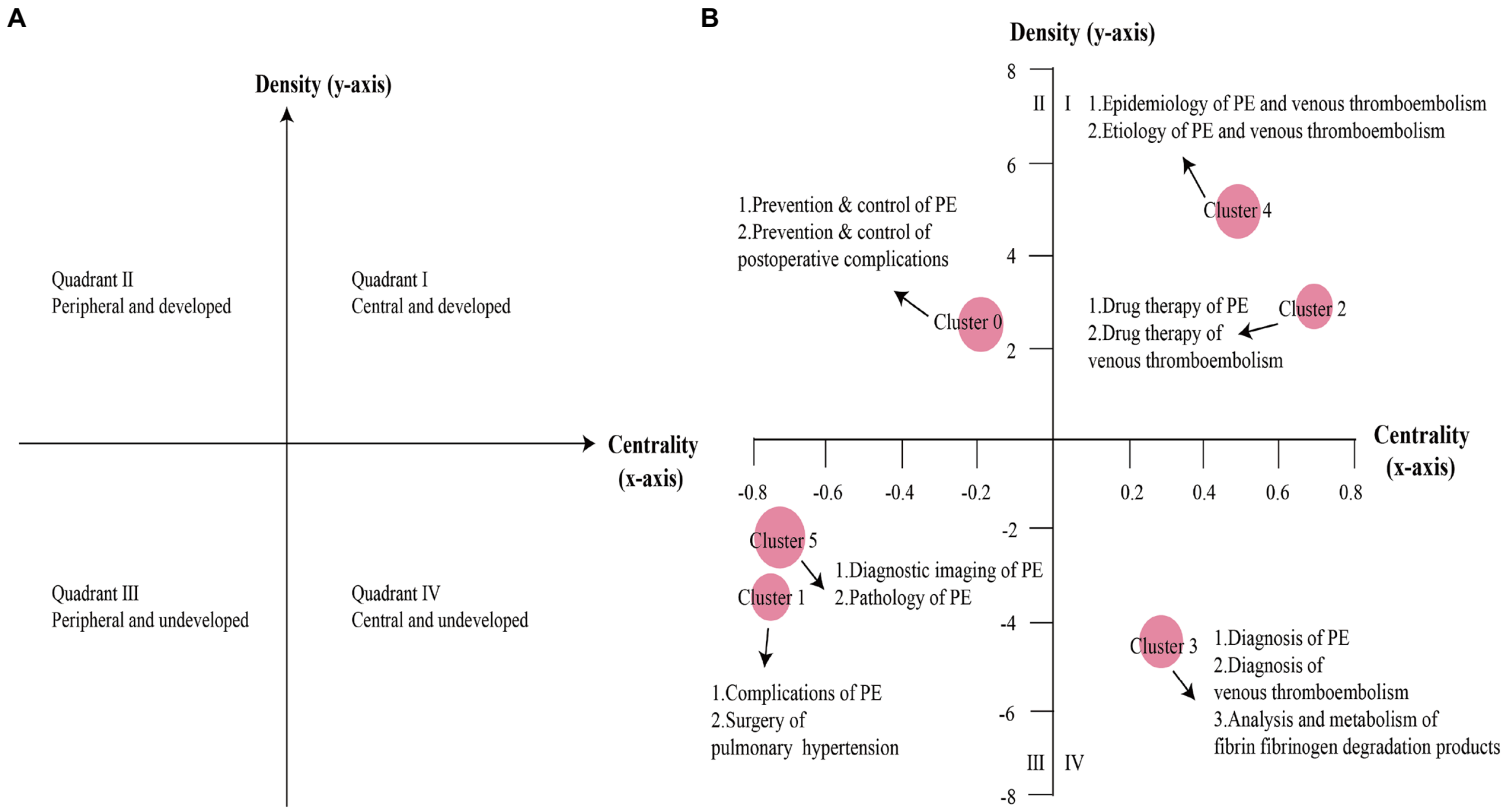
Strategic diagrams for PE. **(A)** The significance of the four quadrants in the strategic diagram. **(B)** Strategic diagram of PE from 2017 to 2021. The main MeSH terms/MeSH subheadings were represented by nodes in different quadrants.

The clusters in Quadrant I show key themes with strong centrality and high density, representing strong ties to other clusters, strong internal interactions, and high levels of development. Quadrant II contains themes that are considered high density and low centrality, which are well developed internally but show insufficient external interactions. Quadrant III describes themes with low density and insufficient centrality; this segment is at the edge of the research field and the research is not yet mature, so these themes are usually considered to be disappearing or emerging. Quadrant IV represents themes that are more central but less internally mature. Based on the calculated density and centrality, themes are depicted as spheres in different Quadrants, representing their internal and external cohesion, respectively, in the strategic diagram. Cluster 2 and Cluster 4 are in Quadrant I, representing the epidemiology, etiology, and clinical pharmacotherapy of PE and VTE at the core of high density and strong centrality. Cluster 0 is in Quadrant II, implying the prevention and control of PE and postoperative complications. Although well developed, Cluster 0 is still at the periphery of the PE research field. Cluster 1 and Cluster 5 are in quadrant III, including immature research on diagnostic imaging, pathology, complications of PE, and surgical treatment of pulmonary hypertension, which are at the edge of the research field and have potential research space. Cluster 3 is in quadrant IV, indicating lack of in-depth research on the diagnosis of PE and VTE, analysis, and metabolism of fibrin/fibrinogen degradation products. Figures 3A,B show the development and trends of each PE theme cluster over the 5-year period.

### Knowledge structure of PE

**Figure 4:**
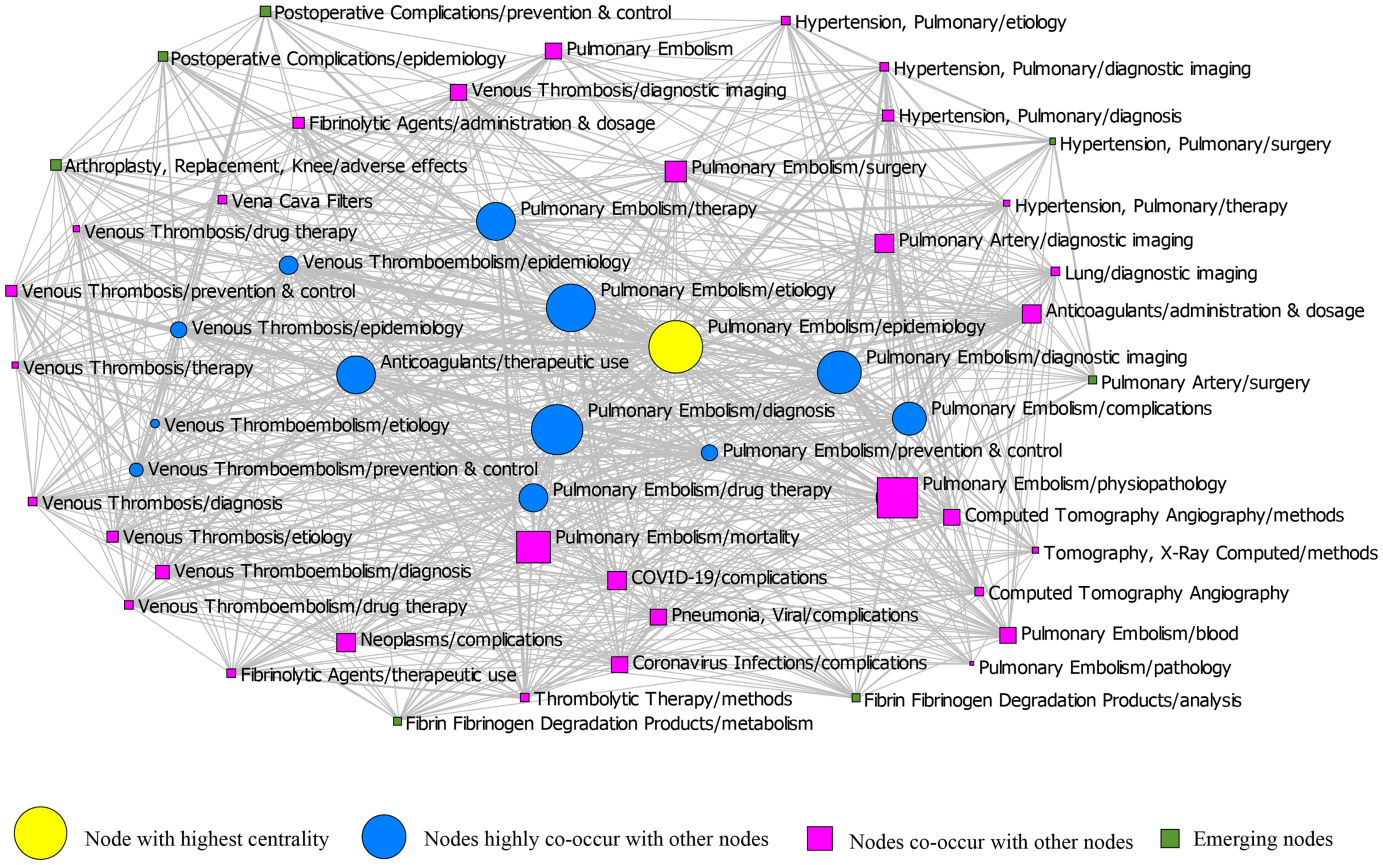
Social network structure map for main Medical Subject Heading (MeSH) terms/subheadings for PE-related publications. Social network analysis for 52 main MeSH terms/MeSH subheadings from 2017 to 2021. Nodes represent MeSH terms/MeSH subheading words, while node size and location indicate the centrality of a word in the social network analysis map. Links represent the connection between two words, and the number or thickness of the lines indicates the co-occurrence frequency of the main MeSH terms/MeSH subheadings pairs.

The social network analysis of PE is shown in Supplementary Additional Table 2 and Table 2, where degree, closeness, and betweenness are all centrality parameters. Degree centrality is the number of other points in a network that are directly connected to a point, and a high number indicates a strong connection with other points, reflecting the centrality of the point in the network. Betweenness centrality measures the extent to which a point is located in the “middle” of other points in the graph, indicating the degree of control of a node over the resources in the network, and the higher the betweenness centrality, the node plays a key intermediary role. Closeness centrality is the sum of the shortest distance from a node to other nodes, and the smaller the proximity centrality, the stronger the node’s ability to be free from control by other nodes. In this study, the social network analysis network was constructed with betweenness centrality. The line thickness represents the co-occurrence frequency, and the node size is proportional to the betweenness centrality of the main MeSH terms/MeSH subheading (Figure 4). The yellow circles represent the nodes with the highest betweenness centrality, which means that “PE/epidemiology” is located at the center of the network and plays a key mediating role in the entire network. The mean degree of centrality in this network is 273.577 ± 203.421 (Table 2), and the blue circles represent nodes with above-average degree centrality, meaning that nodes in this category have more frequent co-occurrence with other nodes, such as “PE/etiology,” “PE/diagnosis,” “PE/therapy,” “PE/complications,” “PE/prevention and control,” “Anticoagulants/therapeutic use.” In addition, the green boxes represent emerging hotspots of PE, including “Postoperative Complications/prevention and control,” “Postoperative Complications/epidemiology,” “Hypertension, Pulmonary/surgery,” “Pulmonary Artery/surgery,” “fibrin/fibrinogen Degradation Products/analysis,” “fibrin/fibrinogen Degradation Products/metabolism,” and “Arthroplasty, Replacement, Knee/adverse effects.”

**Table 2:** Descriptive statistics for centrality measures for PE.

Centralization	Min.	Max.	Mean ± SD
Degree	35.000	919.000	273.577 ± 203.421
Betweenness	0.855	35.884	10.212 ± 9.023
Closeness	33.000	50.000	40.788 ± 4.398

## Discussion

The incidence of PE has shown an increasing trend over the past few decades due to progressive population aging, increased risk of developing VTE, and the increased sensitivity and availability of imaging tests (12, 13). In the United States, the estimated annual incidence of acute PE is 115 cases per 100,000 population (14). In addition, numerous studies have reported that Coronavirus Disease 2019 (COVID-19) may increase the risk of PE (15, 16). The disease burden of PE is bound to receive increasing attention. Due to the increasing volume of PE literature, it is necessary to sort out the knowledge structure and research trends of PE in order to provide researchers and clinicians with a better understanding of the disease. This is the first study aiming to objectively analyze the research characteristics and hotspots of PE from a bibliometric perspective, with the aim of identifying future research directions on the subject of PE.

A total of 5,583 publications about PE were found in the PubMed database for this study. Analysis showed some fluctuations in the number of articles published annually about PE during the 5-year period, with the most articles published in 2020 and the least in 2021. Among the top 15 journals, Thromb Res led the way, along with other major journals such as BMJ Case Rep, Medicine (Baltimore), and J Thromb Haemost. Klok FA, from the Department of Thrombosis and Hemostasis, Leiden University Medical Center, authored the most PE-related publications during this time. Dr. Klok and his team published many valuable results on the epidemiology, diagnosis, treatment, and prognosis of PE.

**Figure 5:**
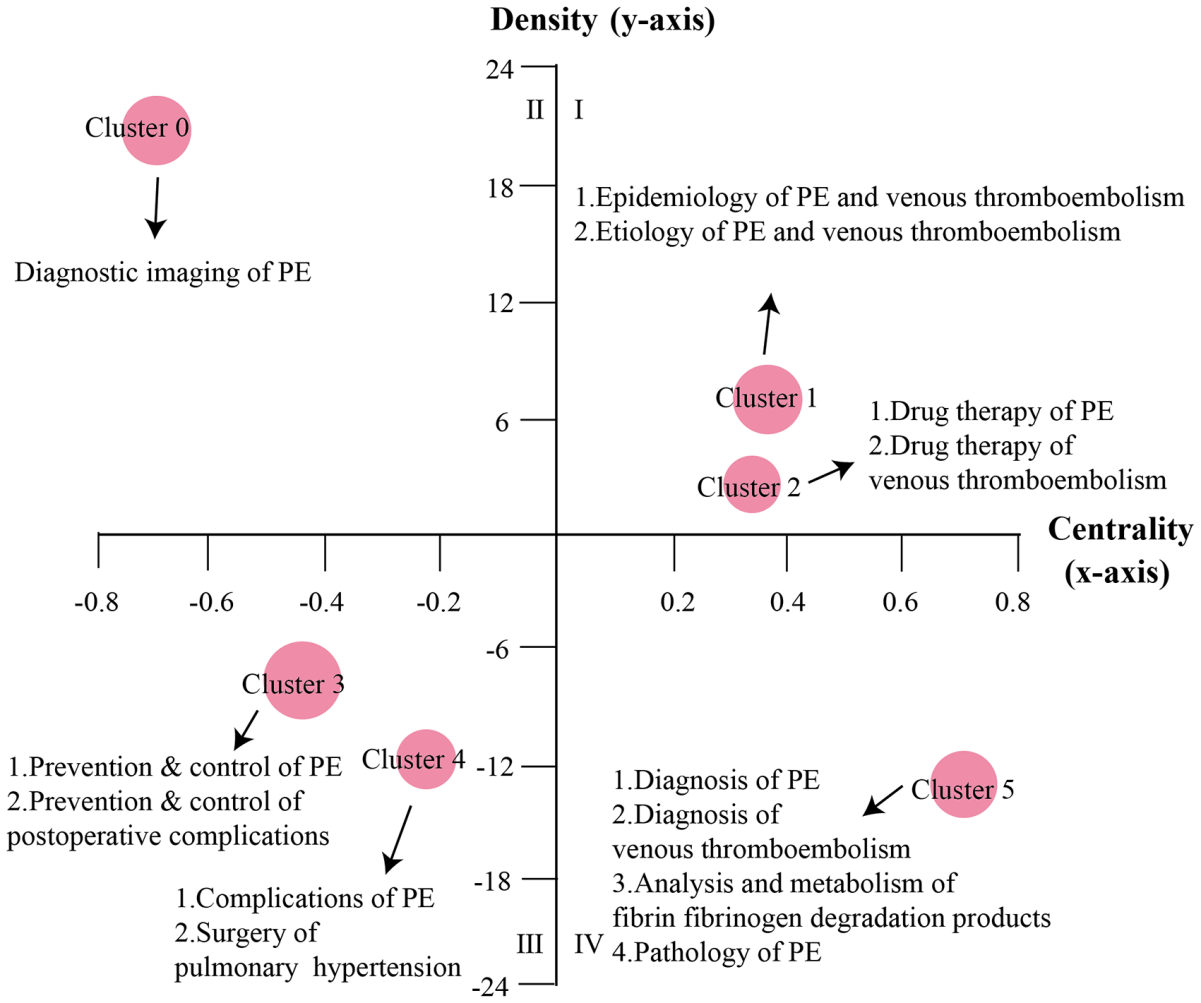
Strategic diagram of PE from 2012 to 2016.

This study combined social network analysis with co-word analysis for in-depth evaluation of the basic knowledge structure of PE. Co-word analysis allows for clustering of closely related MeSH words. The strategic diagram was used to analyze the theme trends of PE. To more prominently show the change in theme trends in PE in the last 5 years, we compared it with previous studies. We collected articles from 2012 to 2016 under the same search criteria and made a strategic diagram after clustering analysis of high-frequency main MeSH terms/MeSH subheadings (Figure 5). It is worth noting that since the clustering ordinal numbers were automatically generated by the gCLUTO software, the content represented by the clustering ordinal numbers in Figure 5 may be different from Figure 3, and we mainly focus on the theme content of each quadrant. Through clustering analysis and strategic diagram analysis, we found that the epidemiology, etiology, and drug therapy of PE and VTE have been in the first quadrant for the past decade, indicating that these themes are not only the most popular but also have received relatively stable attention. With approximately 10 million people affected by VTE worldwide each year and with the annual incidence of acute VTE four times higher in high-income countries than in low-income countries, VTE is a significant contributor to the global burden of disease (17, 18). The high incidence of PE has also been a cause for concern, and likewise, the drug therapy of PE is a topic that is often explored. Prevention and control of PE and postoperative complications changed from being in the third quadrant to the second quadrant, indicating increased attention to the topic and maturing research within the theme. However, preventive management of VTE has been reported to be inadequate in a significant proportion of patients (19, 20), so attention is still needed. The change of diagnostic imaging of PE from being located in the second quadrant to the third quadrant and the pathology of PE from being located in the fourth quadrant to the third quadrant indicates that these two themes are at the edge of the research field in the last 5 years, and the research is still immature and has potential research space. Complications of PE and surgery of pulmonary hypertension have been in the third quadrant for nearly a decade. Post-PE syndrome is an important long-term complication of VTE that reduces the quality of life and causes a significant financial and medical burden (21). The most severe manifestation of post-PE syndrome is chronic thromboembolic pulmonary hypertension, which affects 3% of patients with PE, and almost half of these patients develop functional and motor limitations within 1 year after PE (22, 23). These themes are in the third quadrant and need further study, both in terms of theme depth and closeness to other themes. Regarding the diagnosis of PE and VTE, analysis and metabolism of fibrin/fibrinogen degradation products, this group of topics has been located in the fourth quadrant, suggesting that this group is not sufficiently studied internally.

In social network analysis, “PE/epidemiology” was found to lead the field of PE, as the node is in a pivotal position and plays a key mediating role, according to the value of its betweenness centrality parameter, which is essential to maintain the stability of the entire network. In terms of degree centrality, “PE/diagnostic imaging,” “PE/diagnosis,” “PE/therapy,” “PE/etiology,” “PE/drug therapy,” “PE/epidemiology,” “PE/complications,” “Anticoagulants/therapeutic use,” “PE/prevention and control,” “VTE/epidemiology,” “Venous Thrombosis/epidemiology,” “VTE/prevention and control,” and “VTE/etiology” are in the middle of the network, showing a clear and direct connection with other nodes. Surgical treatment of pulmonary hypertension, prevention and control of postoperative complications, and metabolism and analysis of fibrin/fibrinogen degradation products are at the edge of the network, with both degree centrality, betweenness centrality, and closeness centrality below the mean, indicating that such nodes are both less connected to other nodes and have potential research space. Furthermore, by reviewing the literature, we found a gradual increase in the last 5 years on these marginal topics, such as the literature related to surgical treatment of pulmonary hypertension, from 71 articles in 2017 to 105 articles in 2021. Last but not least, combined with the clinical experience of the researchers, clinicians are placing increasing emphasis on topics such as surgical treatment of pulmonary hypertension and prevention and control of postoperative complications, all of which illustrate that these types of topics represent emerging research hotspots.

## Strengths and limitations

This is the first study to apply bibliometrics for an objective analysis of PE research hotspots and the development of PE to provide researchers and clinicians with an intuitive knowledge structure for future research directions. However, some limitations of our study should be acknowledged. First, only journal articles were included in this study. Secondly, only articles published in English language were included. Third, the co-word analysis is based on high-frequency MeSH terms, and the number of high-frequency MeSH words may have some impact on the results, which may ignore the emerging topics with lower attention and frequency. Fourth, our research work ended at the end of 2021, and the retrieved data cannot be updated in time because the database is dynamically updated. To better guide the research direction, future studies should include multiple databases, multiple languages, and multiple literature types.

## Conclusion

In summary, we applied bibliometrics to objectively analyze the knowledge structure and research trends in PE, using a combination of co-word analysis, biclustering analysis, strategic diagram analysis, and social network analysis. Analysis of PE research trends may help researchers and clinicians to start new studies. We identified potential research space for relatively less explored topics, such as diagnosis, diagnostic imaging, complications, and pathology of PE. In addition, surgical treatment of pulmonary hypertension, prevention and control of postoperative complications, and metabolism and analysis of fibrin/fibrinogen degradation products were identified as emerging research hotspots. Future research projects should focus on unexplored topics and emerging hotspots.

## Data availability statement

The original contributions presented in the study are included in the article/Supplementary material, further inquiries can be directed to the corresponding author.

## Author contributions

JF collected the data and participated in manuscript writing. XX conceived this study and participated in manuscript writing. LZ critically revised the manuscript and provided guidance. All authors read and approved the final manuscript.

## Funding

This study was supported by Liaoning Provincial Natural Science Foundation Project (2022-YGJC-67).

## Conflict of interest

The authors declare that the research was conducted in the absence of any commercial or financial relationships that could be construed as a potential conflict of interest.

## Publisher’s note

All claims expressed in this article are solely those of the authors and do not necessarily represent those of their affiliated organizations, or those of the publisher, the editors and the reviewers. Any product that may be evaluated in this article, or claim that may be made by its manufacturer, is not guaranteed or endorsed by the publisher.

## Supplementary material

The Supplementary material for this article can be found online at: https://www.frontiersin.org/articles/10.3389/fmed.2023.1052928/full#supplementary-material
