# Supplementary material for: A bibliometric analysis of the theme trends and knowledge structures of pulmonary embolism from 2017 to 2021
Source: Front Med (Lausanne). 2023 Feb 23;10:1052928. doi: 10.3389/fmed.2023.1052928 (PMC9997633; doi:10.3389/fmed.2023.1052928)
Supplement: Supplementary file 1 [file Table_1.DOCX]

Supplementary Material

Additional Table 1. High-frequency main MeSH terms/subheadings from the included articles on PE.

| Rank | main MeSH terms/subheadings | Frequency | Proportion  of frequency(%) | Cumulative  Percentage(%) |
| --- | --- | --- | --- | --- |
| 1 | PE/diagnostic imaging | 812 | 3.8628 | 3.8628 |
| 2 | PE/diagnosis | 686 | 3.2634 | 7.1262 |
| 3 | PE/therapy | 503 | 2.3928 | 9.5191 |
| 4 | PE/etiology | 460 | 2.1883 | 11.7073 |
| 5 | PE/drug therapy | 448 | 2.1312 | 13.8385 |
| 6 | PE/epidemiology | 429 | 2.0408 | 15.8794 |
| 7 | PE/complications | 388 | 1.8458 | 17.7251 |
| 8 | Anticoagulants/therapeutic use | 266 | 1.2654 | 18.9905 |
| 9 | PE | 224 | 1.0656 | 20.0561 |
| 10 | PE/prevention & control | 219 | 1.0418 | 21.0979 |
| 11 | VTE/epidemiology | 211 | 1.0038 | 22.1017 |
| 12 | PE/surgery | 192 | 0.9134 | 23.0151 |
| 13 | Venous Thrombosis/epidemiology | 169 | 0.8040 | 23.8190 |
| 14 | PE/mortality | 165 | 0.7849 | 24.6040 |
| 15 | VTE/prevention & control | 158 | 0.7516 | 25.3556 |
| 16 | PE/physiopathology | 149 | 0.7088 | 26.0644 |
| 17 | Thrombolytic Therapy/methods | 146 | 0.6945 | 26.7590 |
| 18 | Pulmonary Artery/diagnostic imaging | 135 | 0.6422 | 27.4012 |
| 19 | Computed Tomography Angiography/methods | 134 | 0.6375 | 28.0386 |
| 20 | VTE/etiology | 128 | 0.6089 | 28.6475 |
| 21 | PE/blood | 127 | 0.6042 | 29.2517 |
| 22 | Vena Cava Filters | 121 | 0.5756 | 29.8273 |
| 23 | Venous Thrombosis/etiology | 104 | 0.4947 | 30.3221 |
| 24 | Hypertension, Pulmonary/therapy | 103 | 0.4900 | 30.8120 |
| 25 | COVID-19/complications | 102 | 0.4852 | 31.2973 |
| 26 | VTE/drug therapy | 101 | 0.4805 | 31.7777 |
| 27 | Anticoagulants  /administration & dosage | 96 | 0.4567 | 32.2344 |
| 28 | Venous Thrombosis/prevention & control | 96 | 0.4567 | 32.6911 |
| 29 | VTE/diagnosis | 88 | 0.4186 | 33.1097 |
| 30 | Fibrinolytic Agents/therapeutic use | 83 | 0.3948 | 33.5046 |
| 31 | Fibrinolytic Agents/  administration & dosage | 83 | 0.3948 | 33.8994 |
| 32 | Venous Thrombosis/drug therapy | 82 | 0.3901 | 34.2895 |
| 33 | Hypertension, Pulmonary/  diagnostic imaging | 82 | 0.3901 | 34.6796 |
| 34 | Postoperative Complications/  epidemiology | 77 | 0.3663 | 35.0459 |
| 35 | Pneumonia, Viral/complications | 76 | 0.3615 | 35.4074 |
| 36 | Hypertension, Pulmonary/surgery | 76 | 0.3615 | 35.7690 |
| 37 | Hypertension, Pulmonary/diagnosis | 75 | 0.3568 | 36.1258 |
| 38 | Tomography, X-Ray Computed/  methods | 75 | 0.3568 | 36.4826 |
| 39 | Hypertension, Pulmonary/etiology | 74 | 0.3520 | 36.8346 |
| 40 | Coronavirus Infections/complications | 74 | 0.3520 | 37.1866 |
| 41 | Computed Tomography Angiography | 73 | 0.3473 | 37.5339 |
| 42 | Neoplasms/complications | 71 | 0.3378 | 37.8717 |
| 43 | Venous Thrombosis/  diagnostic imaging | 71 | 0.3378 | 38.2094 |
| 44 | fibrin/fibrinogen Degradation Products/analysis | 66 | 0.3140 | 38.5234 |
| 45 | Arthroplasty, Replacement, Knee/  adverse effects | 64 | 0.3045 | 38.8278 |
| 46 | Venous Thrombosis/diagnosis | 62 | 0.2949 | 39.1228 |
| 47 | Lung/diagnostic imaging | 61 | 0.2902 | 39.4130 |
| 48 | PE/pathology | 59 | 0.2807 | 39.6936 |
| 49 | Pulmonary Artery/surgery | 58 | 0.2759 | 39.9696 |
| 50 | Venous Thrombosis/therapy | 57 | 0.2712 | 40.2407 |
| 51 | fibrin/fibrinogen Degradation Products/metabolism | 54 | 0.2569 | 40.4976 |
| 52 | Postoperative Complications/  prevention & control | 53 | 0.2521 | 40.7497 |

Additional Table 2. Individual centrality of PE.

| Rank | Main MeSH terms/MeSH subheadings | Degree | Betweenness | Closeness |
| --- | --- | --- | --- | --- |
| 1 | PE/diagnostic imaging | 919 | 29.67 | 48.5 |
| 2 | PE/diagnosis | 803 | 33.311 | 49.5 |
| 3 | PE/therapy | 663 | 25.429 | 48 |
| 4 | PE/etiology | 685 | 31.726 | 49 |
| 5 | PE/drug therapy | 639 | 17.889 | 46 |
| 6 | PE/epidemiology | 778 | 35.884 | 50 |
| 7 | PE/complications | 502 | 22.651 | 46.5 |
| 8 | Anticoagulants/therapeutic use | 515 | 24.964 | 47.5 |
| 9 | PE | 137 | 8.849 | 37 |
| 10 | PE/prevention & control | 464 | 10.326 | 44 |
| 11 | VTE/epidemiology | 497 | 10.920 | 44 |
| 12 | PE/surgery | 210 | 13.540 | 42.5 |
| 13 | Venous Thrombosis/epidemiology | 423 | 10.443 | 43.5 |
| 14 | PE/mortality | 179 | 19.459 | 45.5 |
| 15 | VTE/prevention & control | 357 | 7.957 | 41.5 |
| 16 | PE/physiopathology | 222 | 27.430 | 47.5 |
| 17 | Thrombolytic Therapy/methods | 268 | 4.345 | 39 |
| 18 | Pulmonary Artery/diagnostic imaging | 261 | 12.046 | 41.5 |
| 19 | Computed Tomography Angiography/methods | 224 | 9.312 | 40.5 |
| 20 | VTE/etiology | 306 | 4.459 | 40 |
| 21 | PE/blood | 165 | 9.776 | 41 |
| 22 | Vena Cava Filters | 198 | 4.377 | 39.5 |
| 23 | Venous Thrombosis/etiology | 250 | 6.429 | 41 |
| 24 | Hypertension, Pulmonary/therapy | 216 | 3.466 | 36 |
| 25 | COVID-19/complications | 207 | 10.924 | 44 |
| 26 | VTE/drug therapy | 242 | 3.685 | 40 |
| 27 | Anticoagulants/administration & dosage | 208 | 11.131 | 43.5 |
| 28 | Venous Thrombosis/prevention & control | 262 | 6.256 | 40.5 |
| 29 | VTE/diagnosis | 230 | 8.481 | 42.5 |
| 30 | Fibrinolytic Agents/therapeutic use | 157 | 3.639 | 38.5 |
| 31 | Fibrinolytic Agents/administration & dosage | 150 | 6.472 | 38 |
| 32 | Venous Thrombosis/drug therapy | 207 | 2.542 | 38.5 |
| 33 | Hypertension, Pulmonary/diagnostic imaging | 157 | 4.544 | 36.5 |
| 34 | Postoperative Complications/epidemiology | 120 | 2.906 | 35.5 |
| 35 | Pneumonia, Viral/complications | 183 | 9.781 | 41.5 |
| 36 | Hypertension, Pulmonary/surgery | 151 | 1.630 | 33 |
| 37 | Hypertension, Pulmonary/diagnosis | 183 | 5.926 | 37 |
| 38 | Tomography, X-Ray Computed/methods | 111 | 2.604 | 35 |
| 39 | Hypertension, Pulmonary/etiology | 185 | 3.996 | 35.5 |
| 40 | Coronavirus Infections/complications | 177 | 9.554 | 41 |
| 41 | Computed Tomography Angiography | 134 | 4.302 | 38 |
| 42 | Neoplasms/complications | 154 | 10.862 | 42.5 |
| 43 | Venous Thrombosis/diagnostic imaging | 123 | 9.524 | 40 |
| 44 | fibrin/fibrinogen Degradation Products/analysis | 117 | 2.213 | 37.5 |
| 45 | Arthroplasty, Replacement, Knee/adverse effects | 113 | 4.902 | 37.5 |
| 46 | Venous Thrombosis/diagnosis | 171 | 4.354 | 40 |
| 47 | Lung/diagnostic imaging | 105 | 5.183 | 37.5 |
| 48 | PE/pathology | 35 | 0.855 | 33.5 |
| 49 | Pulmonary Artery/surgery | 123 | 1.604 | 34 |
| 50 | Venous Thrombosis/therapy | 129 | 3.129 | 39.5 |
| 51 | fibrin/fibrinogen Degradation Products/metabolism | 86 | 1.360 | 36 |
| 52 | Postoperative Complications/prevention & control | 125 | 3.984 | 35.5 |
